# The Impact of Pain, Threat Perception and Emotional Distress on Suicide Risk in Individuals with Colorectal Cancer

**DOI:** 10.3390/nursrep14040194

**Published:** 2024-09-25

**Authors:** Alba Espuig, Maria Pons-Vinent, Eva Carbajo, Laura Lacomba-Trejo

**Affiliations:** 1 Facultat de Psicologia i Logopèdia, Universitat de València, 46010 València, Spain; alesa3@alumni.uv.es (A.E.); vinent@alumni.uv.es (M.P.-V.); 2 Clinical and Health Psychology Unit, Consorcio Hospital General Universitario de València, 46014 València, Spain; carbajo_eva@gva.es; 3 Department of Developmental and Educational Psychology, Facultat de Psicologia i Logopèdia, Universitat de València, 46010 València, Spain

**Keywords:** colorectal cancer, pain, perception of threat, emotional distress, suicide risk, nursing

## Abstract

Background: Colorectal cancer (CRC) can significantly impact mental health, increasing suicide risk. Variables such as pain and threat perception may be crucial. This study aims to identify predictors of suicide risk in individuals with CRC. Methods: A total of 71 participants (76.06% men) aged 27 to 88 years (*M* = 65.18, *SD* = 12.02) were assessed using the SF-36 for pain, the Brief Illness Perception Questionnaire (B-IPQ) for threat perception, the Hospital Anxiety and Depression Scale (HADS) for emotional distress, and the Plutchik suicide risk scale for suicide risk. Descriptive, comparative, and correlational analyses were performed, followed by a linear regression analysis. Results: Nearly 20% of participants exhibited high suicide risk. There was a moderate-to-high association between suicide risk and the perception of threat, pain, and emotional distress. The linear regression model explained 39% of the variance in suicide risk, with threat perception, pain, and emotional distress as significant predictors. Conclusions: These results indicate the need for multidisciplinary care for individuals with CRC, including emotional support from health psychologists, and holistic, human-centered care from nursing and medical professionals. Future research is necessary to further explore these relationships and improve patient care strategies.

## 1. Introduction

Cancer is one of the leading causes of morbidity and mortality worldwide [1,2]. It refers to a group of diseases characterized by uncontrolled and abnormal cell division and growth, due to gene mutations. These cells can form tumors that may be benign or malignant, with the potential to spread to other parts of the body [3]. In 2020, 19.3 million new cases were recorded globally, with colorectal cancer (CRC) being the third most common cancer in both men and women, accounting for 10% of cancer incidence and 9.4% of cancer mortality [4,5].

CRC encompasses cancers located in the large intestine, from the cecum to the rectum. Despite advancements in treatment, which have improved survival rates, CRC patients often face significant psychological challenges [6,7]. In this sense, it is common for people with CRC to experience pain that can have a significant impact on their quality of life, as well as their mental health [8,9]. The prevalence rate of pain in people with CRC is estimated to be more than 70%, depending on the stage of the disease [10]. Pain may be related to the treatment or to the tumor itself and, in fact, it has been seen that after the diagnosis of CRC, 51% of those affected showed pain or fatigue symptoms [11]. Chronic cancer-related pain affects daily activities, relationships, mood, sleep, and overall health, often more severely than the cancer itself [11].

Beyond the physical symptoms associated with cancer, psychological problems are also commonly observed [12]. The stress associated with diagnosis, fear of death, treatment, and prognosis can lead to mental health issues such as anxiety and depression [6,7]. Clinically significant depressive and anxiety symptoms are prevalent among CRC population, with anxiety rates ranging from 1.0% to 47.2% of patients, and depression rates ranging from 1.6% to 57.0% [13]. And, compared to physically healthy individuals, colorectal cancer patients exhibit a roughly 10% higher prevalence of depressive and anxiety symptoms [14]. Anxiety is particularly prevalent while awaiting diagnostic results, and sustained anxiety can lead to depressive symptoms and increased suicide risk, especially in advanced disease stages where physical and mental health decline [15,16].

Suicide is a complex, multifactorial phenomenon often linked to mental health problems, and poses a significant public health issue. Globally, more than 700,000 people die by suicide each year [17]. Cancer has been identified as a factor associated with suicide risk among affected individuals, with poor prognosis, disease progression, symptoms of depression, feelings of helplessness, disturbed interpersonal relationships and uncontrolled pain cited as contributing factors [18]. People with cancer are at double the risk of dying by suicide compared to the general population [19]. Specifically, regarding CRC, numerous studies based on national registries have underscored the risk of suicide within this population [20,21].

Despite the above, the perception of threat from the disease is crucial for adaptive responses, influencing emotional distress and coping mechanisms [22]. The Common-Sense Model of Self-Regulation suggests that individuals create mental representations of their illness based on available information, to manage their condition effectively [23]. These representations are influenced by cultural and social factors, information from significant others, and personal experiences, shaping their perception of the disease and its threat [22,24]. A higher perception of threat is associated with increased distress symptoms and risk of suicide, regardless of the actual threat level of the diagnosis [22].

Given the global impact and increasing incidence of CRC and suicide, it is essential to understand the interplay between pain, threat perception, and emotional distress in this population, to enhance person-centered cancer care through nursing practices. The objective of this study is to identify the predictors of suicide risk in individuals with CRC. The following are expected: Hypothesis 1 (H1) higher levels of pain will be associated with increased emotional distress and risk of suicide in individuals with CRC; Hypothesis 2 (H2) a greater perception of illness threat will be associated with an increased emotional distress and risk of suicide in individuals with CRC; Hypothesis 3 (H3) higher levels of emotional distress will be associated with an increased risk of suicide in individuals with CRC; and Hypothesis 4 (H4) combined, pain, threat perception, and emotional distress will significantly predict suicide risk in individuals with CRC.

## 2. Materials and Methods

### 2.1. Participants

This study included 71 individuals with GI cancer from the Oncology Unit of the Consorcio Hospital General Universitario de Valencia (CHGUV).

Inclusion criteria from the GRAMGEA protocol [25] were the following: (a) undergoing major abdominal surgery and thus not suitable for major ambulatory surgery; (b) aged between 18 and 85 years; (c) adequate cognitive state (able to understand and collaborate); (d) anesthesia risk ≤ II according to the ASA (American Society of Anesthesiologists) scale. Exclusion criteria were (a) requiring urgent surgery or (b) being a pediatric patient.

Additional inclusion criteria were (a) having completed the entire battery of questionnaires; (b) being informed about the study procedure and having signed the informed consent; and (c) being diagnosed with gastrointestinal cancer.

### 2.2. Variables and Instruments

Sociodemographic and clinical variables (marital status, living situation, children, occupation, diagnosis, stage, and hospitalization) were assessed using a custom scale. In addition, the following variables were assessed with questionnaires validated for the study sample:

- **Pain:** evaluated using the Bodily Pain subscale of the Short Form 36 Health Survey (SF-36) [26]. The SF-36 measures various aspects of health-related quality of life, including pain intensity and its limitations. The Bodily Pain subscale is scored from 0 to 100, where higher scores indicate less pain and fewer limitations due to pain. The Cronbach’s alpha for the SF-36 ranges from 0.71 to 0.94 across subscales [27]. In a review with a Spanish population, including cancer patients, the scales exceeded the proposed reliability standard (α ≥ 0.70) [28]. In this study, the observed internal consistency of the bodily pain subscale was α = 0.87.
- **Perception of Threat:** measured using the Brief Illness Perception Questionnaire (B-IPQ) [29,30]. This questionnaire comprises 9 items that evaluate cognitive and emotional perceptions of illness threat. The first 8 items correspond to factors such as consequences, duration, personal control, treatment control, identity, concern, emotional response, and understanding. These are rated on a Likert scale from 0 to 10, with higher scores indicating a greater perception of threat. The final item is an open question about the perceived main causes of the illness. Overall scores are calculated by reversing items 3, 4, and 7, and summing them with items 1, 2, 5, 6, and 8. Higher total scores indicate a greater perceived threat of illness [31,32]. The B-IPQ has shown adequate psychometric properties, with internal consistency indices ranging from α = 0.67 to 0.98 [32]. In this study, Cronbach’s alpha was α = 0.67.
- **Emotional Distress:** assessed using the Hospital Anxiety and Depression Scale (HADS) [33]. This scale measures anxiety and depression symptoms without considering somatic symptoms, making it suitable for individuals with medical diagnoses. The HADS comprises 14 items forming two subscales: anxiety (HADS-A) and depression (HADS-D). Each item is rated on a Likert scale from 0 (minimum) to 3 (maximum presence of symptoms). Items 1, 3, 6, 8, 10, 11, and 13 are reversed. Total scores for each subscale indicate levels of anxiety and depression. Additionally, a total Emotional Distress score can be obtained by summing both subscales. The interpretation of HADS scores is as follows: 0–7 indicates normal anxiety and depression symptoms, 8–10 a probable case of anxiety or depression, and >10 a clinical problem. Regarding emotional distress, ≥20 scores indicated a clinical problem [34]. For this study, Cronbach’s alpha was α = 0.90.
- **Suicide Risk:** assessed using the Plutchik Suicide Risk Scale [35,36]. This instrument measures the level of suicide risk and feelings related to depression and hopelessness. It consists of 15 dichotomous items (yes/no), with one point awarded for each affirmative answer, resulting in a maximum score of 15. Higher scores indicate a higher risk of suicide, with scores of six or more indicating significant risk [37]. The Spanish version demonstrated adequate psychometric properties, with an internal consistency of α = 0.90 [37,38]. In this study, Cronbach’s alpha was α = 0.71.

### 2.3. Procedure

Participants were informed about the study and completed the consent form before the administration of the questionnaire battery. Assessments were conducted in the Health Psychology Unit of CHGUV between 2019 and 2024. Participants were referred by the General and Digestive Surgery Service under the GRAMGEA protocol [25]. GRAMGEA (Grupo de Rehabilitación Multimodal del hospital General) is a multidisciplinary team that includes personnel from surgery, anesthesiology, endocrinology–nutrition, rehabilitation, psychology, nursing, auxiliary services, and administrative staff. The main objective of GRAMGEA is to ensure that patients are in optimal functional and nutritional condition for surgery.

### 2.4. Design

The study used a non-experimental, cross-sectional design, collecting data at a single time point for exploratory and descriptive purposes.

### 2.5. Analysis

Data analysis was performed using SPSS 28.0. Descriptive analyses described the sample based on sociodemographic and clinical variables. Pearson correlations were conducted to examine relationships between the variables and linear regressions to predict suicide risk in people with CRC cancer through perceived threat of illness, pain and emotional distress.

## 3. Results

### 3.1. Descriptive Analysis

According to the sociodemographic and clinical characteristics of the sample, participants were aged between 27 and 88 years (*M* = 65.18, *SD* = 12.02), with 76.06% men and 23.94% women. The majority of the sample (50.09%) were married, 24.24% were widowed, 15.15% were divorced and 1.52% were single. Of the total sample, 15.38% lived alone and the remaining 84.62% lived with someone, and 78.33% had children. Regarding employment status, the majority were retired (50.77%), a smaller percentage were on temporary leave (12.31%) and the rest were employed (9.23%), dedicated to housework duties (9.23%), unemployed (7.69%) or on permanent disability allowance (4.62%). Study participants had a diagnosis of colon cancer (76.05%) or rectal cancer (23.94%). The oncologists from the GRAMGEA group evaluated the participants and determined the stage of colorectal cancer based on the TNM system of the American Joint Committee on Cancer (AJCC). Thus, the majority were in stage 2 (44.62%) or 3 (32.30%), a smaller percentage in stage 1 (18.4%) and the remaining participants in stage 4 (3.08%) or 0 (1.54%). Of the total sample, 20.75% had been hospitalized, while the remainder had not (Table 1).

In relation to pain, moderate-to-high levels were observed (*M* = 31.56, *SD* = 33.51), considering that lower scores indicate greater pain and more limitations. A total of 63.60% of the sample demonstrated impairment, with an impact below 50%.

In relation to threat perception, most of the obtained values were low to moderate (*M* = 33.30; *SD* = 13.28). It was observed that the following factors were particularly relevant: low personal control over the disease and as low understanding, the associated concerns, and the emotional impact. Conversely, participants reported perceiving an improvement in the disease due to treatment (Table 2).

Regarding emotional distress, moderate levels were observed within the participants (*M* = 10.77; *SD* = 7.37). A total of 13.04% of the sample showed clinically significant emotional distress.

In terms of suicide risk, participants generally showed low scores (*M* = 2.63; *SD* = 2.45). However, 16.50% exhibited a total score above 6, indicating a high suicide risk.

### 3.2. Correlational Analysis

Pearson correlations were conducted to examine relationships between suicide risk, pain, threat perception and emotional distress. Significant and positive correlations were found between suicide risk and all variables evaluated: pain (*rx* = 0.51; *p* ≤ 0.01), threat perception (*rx* = 0.45; *p* ≤ 0.01), and emotional distress (*rx* = 0.60; *p* ≤ 0.01). Positive significant correlations were also shown between pain and threat perception (*rx* = 0.46; *p* ≤ 0.01), pain and emotional distress (*rx* = 0.40; *p* ≤ 0.01), and threat perception and emotional distress (*rx* = 0.49; *p* ≤ 0.01) (Table 3).

### 3.3. Predictive Analysis

A stepwise linear regression was conducted to predict suicide risk, based on pain, illness threat perception, and emotional distress. The analysis was performed in two steps.

In the first step, pain and illness threat perception were entered as predictors. The results showed that these two variables together accounted for 28.80% of the variance in suicide risk, and that the inclusion of these variables was significant (*p* < 0.001). In the second step, emotional distress was added as a predictor. This addition improved the model significantly, explaining 39.40% of the variance in suicide risk. This final model was also significant (*p* < 0.001). These results suggest that pain, illness threat perception, and emotional distress are significant predictors of suicide risk in individuals with CRC, highlighting the importance of comprehensive emotional and psychological support for this population (Table 4).

## 4. Discussion

The findings of the study largely align with our hypotheses, highlighting the significant impact of pain, illness threat perception and emotional distress on suicide risk among CRC patients. Consistent with H1, the results indicate that higher levels of pain are significantly associated with increased emotional distress and, consequently, an elevated risk of suicide. This finding underscores the debilitating effect of pain on the psychological well-being of cancer patients. Chronic pain can severely impair daily functioning and quality of life, contributing to feelings of hopelessness and despair that heighten suicide risk. These results support previous research, indicating that cancer-related pain detrimentally impacts the mental health of afflicted individuals [9] and that an increase in pain intensity exacerbates depressive symptoms [39], emphasizing the need for effective pain management as a critical component of comprehensive cancer care [8,9].

Supporting H2, the study found that a greater perception of illness threat is associated with increased emotional distress and a higher risk of suicide. The Common-Sense Model of Self-Regulation suggests that individuals’ mental representations of their illness, shaped by personal experiences and social influences, significantly impact their emotional responses [23,24]. In the context of CRC, people who perceive their illness as highly threatening are more likely to experience severe emotional distress, which can exacerbate suicidal ideation and behaviors. These findings align with the existing literature, which posits that illness representations are related to illness outcomes in individuals with cancer, specifically correlating with elevated levels of psychological distress and diminished levels of functioning and quality of life [40] and highlighting the importance of addressing patients’ perceptions and fears through targeted psychological interventions. Interventions that aim to reshape illness perceptions and provide coping strategies can help reduce emotional distress and suicide risk in these patients.

H3 posited that higher levels of emotional distress would be directly associated with an increased risk of suicide. The results confirmed this hypothesis, indicating that emotional distress is a significant predictor of suicide risk in CRC patients. The existing literature identifies anxiety and depression as prevalent issues among cancer patients, particularly those with a poor prognosis or advanced disease stages [7,41]. The outcomes in this study are consistent with previous research that has pointed out psychological distress (including depression and anxiety) as a consistent predictor of suicide in individuals with cancer [42]. The strong association between these variables underscores the necessity for routine psychological screening and support for CRC patients throughout their treatment journey. Implementing regular mental health assessments and providing access to mental health professionals should be a standard part of oncological care.

As anticipated in H4, the combined model of pain, illness threat perception, and emotional distress significantly predicted suicide risk in individuals with CRC. Notably, emotional distress emerged as the strongest predictor within the model, indicating its central role in mediating the effects of pain and threat perception on suicide risk. These findings suggest that while pain and threat perception are critical factors, their impact on suicide risk is largely mediated through emotional distress. This integrated understanding calls for a holistic approach to patient care that simultaneously addresses physical symptoms, cognitive perceptions, and emotional well-being.

The role of nursing in cancer patients’ care is increasingly recognized, enhancing the likelihood of improving treatment and reducing anxiety [43]. Recently, the role of the Clinical Nurse Specialist (CNS) has expanded as that of advanced nursing practice [44], significantly improving CRC patient outcomes by providing psychological support, offering information, assisting in symptom management, and improving service delivery, thereby increasing overall patient satisfaction [45]. Nurses fulfill a bridging role between the patient, family, and other healthcare professionals such as physicians; thus, the development of effective communication skills and multidisciplinary collaboration are essential key components [46].

Nurses are uniquely positioned to address both the physical and psychological needs of patients. They are often the first to identify signs of psychological distress, such as anxiety and depression, which are prevalent among cancer patients and are significant predictors of suicide risk. By providing emotional support and counseling, nurses help mitigate the effects of pain and the perception of illness threat, which are strongly associated with emotional distress.

Moreover, nursing interventions that focus on educating patients about their condition and treatment options empower them, reduce their perceived threat, and improve their overall mental health. As coordinators of care, nurses ensure that patients receive timely and comprehensive support from various healthcare providers, facilitating multidisciplinary collaboration that is crucial in managing chronic conditions like colorectal cancer. This holistic approach to care, which integrates physical, cognitive, and emotional aspects, aligns with the central tenets of the nursing metaparadigm and underscores the importance of nursing in not only enhancing the quality of life, but also in preventing severe outcomes such as suicide in individuals with chronic diseases.

Despite the valuable insights gained, this study has several limitations. In the first place, the study had a cross-sectional design, which limited the possibility of establishing causal relationships. Therefore, future longitudinal studies are needed to confirm the predictive relationships and explore potential mediators and moderators. Additionally, the sample size was relatively small, and all participants belonged to the same hospital center (monocentric study), which may not be representative of the broader CRC population. The sample also included a higher percentage of men (76.06%), potentially affecting the generalizability of the results. Furthermore, while validated questionnaires were used, self-reported measures may be subject to memory bias or social desirability bias. It would be beneficial to complement these data with clinical assessments by health professionals. The study did not consider certain influential factors, such as the type and duration of treatment received, social support, and other socioeconomic factors that could affect suicide risk and emotional well-being. Lastly, the results are based on a specific cultural context (Valencia, Spain), and perceptions of threat, pain, and emotional distress may vary in different cultural settings. Therefore, further research with larger, multicenter, and diverse samples employing probabilistic sampling is warranted to enhance the generalizability of the findings.

In conclusion, this study underscores the critical role of pain, illness threat perception, and emotional distress in predicting suicide risk among CRC patients. Addressing these factors through integrated, multidisciplinary care approaches is essential to improve the mental health and overall well-being of individuals battling colorectal cancer. Within this framework, nurses play a central role in person-centered care. On one hand, they are directly engaged with individuals suffering from cancer, providing essential emotional support and delivering necessary care and information. And on the other hand, they act as intermediaries between cancer patients and other healthcare practitioners, such as physicians and psychologists, thereby fostering collaboration and facilitating multidisciplinary teamwork.

The findings of this study have important clinical implications. First, they highlight the necessity of comprehensive pain-management strategies to alleviate physical suffering and reduce associated emotional distress. Second, they emphasize the importance of psychological interventions aimed at modifying illness perceptions and providing emotional support. Counseling and third-generation therapies, as well as cognitive behavioral therapy, could help individuals with CRC manage the situations they face due to the illness, adjust their expectations and perceptions, and emotionally process the situation. Lastly, the results advocate for regular psychological assessments to identify patients at high risk of suicide and implement timely interventions. Due to the significant role nurses play in the care of individuals with cancer, it is advisable to consider these recommendations. Management of pain, perception of threat, and emotional distress can be alleviated through a multidisciplinary and person-centered nursing approach, with particular emphasis on clinical symptom management, information provision, and emotional support. Therefore, future research focusing on nursing practices aimed at enhancing high-quality care is recommended, in order to assess their impact on the quality of life and well-being of individuals with cancer.

## 5. Conclusions

Our findings indicate that higher levels of pain and greater perception of illness threat are associated with increased emotional distress, which in turn significantly elevates the risk of suicide in this population. Importantly, emotional distress emerged as the strongest predictor of suicide risk, underscoring the need for comprehensive psychological support alongside effective pain management and interventions targeting illness perceptions. Regarding the practical implications of the work, psychological intervention is recommended, to adjust the threat perception of individuals with CRC, as well as to provide psychological support in the form of counseling. Health psychologists play a particularly important role in this aspect. Additionally, nursing professionals are crucial in detecting cases that require prompt and early attention, thereby preventing future complications.

The integration of physical, cognitive, and emotional aspects into patient care is essential for mitigating suicide risk among individuals with CRC. Health professionals such as nurses should prioritize routine psychological assessments and adopt multidisciplinary approaches that address both physical symptoms and psychological well-being. By addressing these multifaceted factors, we can enhance the quality of life and mental health outcomes for individuals with CRC, ultimately reducing the incidence of suicide in this vulnerable population.

## Figures and Tables

**Table 1:** Sociodemographic and clinical variables.

		*n* (%)
Marital status	Bachelor	1 (1.52%)
	Married	39 (59.09%)
	Widow	16 (24.24%)
	Divorced	10 (15.15%)
Living situation	Alone	10 (15.40%)
	Accompanied	55 (84.60%)
Children	Yes	47 (78.33%)
	No	13 (21.67%)
Occupation	Active	6 (9.23%)
	Unemployed	5 (7.69%)
	Temporary leave	8 (12.31%)
	Inability	3 (4.62%)
	Retirement	33 (50.77%)
	Housework	6 (9.23%)
	Unknown	4 (6.5%)
Diagnosis	Colon cancer	54 (76.05%)
	Rectal cancer	17 (23.94%)
Stage	0	1 (1.54%)
	1	12 (18.46%)
	2	29 (44.62%)
	3	21 (32.30%)
	4	2 (3.08%)
Hospitalization	Yes	11 (20.75%)
	No	42 (79.25%)

**Table 2:** Descriptive analysis.

B-IPQ Factors	*M*	*SD*
Consequences	4.51	3.27
Timeline	4.17	2.48
Personal control	6.49	3.50
Treatment control	1.71	1.98
Identity	3.35	3.38
Concern	5.71	3.17
Understanding	2.30	2.84
Emotional response	4.84	3.30

Note: *M* = Mean; *SD* = Standard deviation.

**Table 3:** Correlations between pain, threat perception, emotional distress and suicide risk.

	Pain	Threat Perception	Emotional Distress	Suicide Risk
Pain	1			
Threat perception	0.46 **	1		
Emotional distress	0.40 **	0.49 **	1	
Suicide risk	0.51 **	0.45 **	0.60 **	1

Note: ** *p* ≤ 0.01.

**Table 4:** Hierarchical regression model.

	Suicide Risk in CRC			
Predictors	∆*R*^2^	∆*F*	*β*	*t*
Step 1	0.31 ***	12.73 ***		
Pain			0.30	2.61 *
Illness threat perception			0.27	2.30 *
Step 2	0.11 ***	10.77 **		
Emotional distress			0.34	3.28 **
Durbin–Watson	2.46			
*R* ^2^_adj_	0.39 ***			

Note: ∆*R*^2^ = change in *R*^2^; *R*^2^_adj_ = *R*^2^_adjusted_; *β* = regression coefficient; * *p* < 0.05; ** *p* ≤ 0.01; *** *p* ≤ 0.001.

## Data Availability

Data from the study can be made available upon reasonable request to the corresponding author.

## Public Involvement Statement

No public involvement in any aspect of this research beyond the role of study participants who completed the surveys.

## Guidelines and Standards Statement

This manuscript was drafted against the STROBE (Strengthening the Reporting of Observational Studies in Epidemiology) guidelines for observational research.
